# Drug Crystal-Related Gastrointestinal Complications Involve Crystal-Induced Release of Neutrophil and Monocyte Extracellular Traps

**DOI:** 10.3390/cells9112481

**Published:** 2020-11-15

**Authors:** Tehyung Kim, Sueli de Oliveira Silva Lautenschlager, Qiuyue Ma, Kathrin Eller, Marion Julia Pollheimer, Danielle Lazarin-Bidóia, Celso Vataru Nakamura, Hans-Joachim Anders, Stefanie Steiger

**Affiliations:** 1 Division of Nephrology, Department of Medicine IV, Ludwig-Maximilians-University Hospital Munich, 80336 Munich, Germany; tehyung95@gmail.com (T.K.); qiuyue.ma@med.uni-muenchen.de (Q.M.); 2 Postgraduate Program in Pharmaceutical Sciences, State University of Maringá, Maringá, Paraná 5790, Brazil; lautenschlager@uem.br (S.d.O.S.L.); dlbidoia@gmail.com (D.L.-B.); cvnakamura@uem.br (C.V.N.); 3 Division of Nephrology, Department of Internal Medicine, Medical University Graz, 8036 Graz, Austria; kathrin.eller@medunigraz.at; 4 Diagnostic and Research Institute of Pathology, Medical University of Graz, 8010 Graz, Austria; marion.pollheimer@medunigraz.at

**Keywords:** sevelamer, polystyrene sulfonate, cholestyramine, patiromer, exchange resin, extracellular traps, intestinal necrosis, neutrophils, monocytes

## Abstract

Ion-exchange resins are commonly used to manage complications of chronic kidney disease, such as hyperphosphatemia, hyperkalemia, and hypercholesterolemia. Occasionally, these drugs can irritate the gastrointestinal lining and cause life-threatening intestinal necrosis. Currently, the pathophysiology of drug crystal-induced intestinal necrosis is not well understood. We hypothesized that crystals of ion-exchange resins like sevelamer, polystyrene sulfonate, and cholestyramine can trigger the formation of neutrophil and monocyte extracellular traps by contributing to intestinal barrier dysfunction. Light and fluorescence microscopy of the colonic resection specimen from a patient with chronic kidney disease revealed severe intestinal necrosis, ulceration, sevelamer crystals, and inflammation upon oral intake of sevelamer, as well as the formation of neutrophil extracellular traps in proximity to small sevelamer crystals. Indeed, drug crystals reduced metabolic activity and induced barrier dysfunction and cell death in human intestinal epithelial cells in vitro. In addition, drug crystals triggered the release of neutrophil and monocyte extracellular traps. Taken together, these data raise the possibility that besides other factors including chronic kidney disease, diabetes mellitus, and hypertension, drug crystals may further amplify a pre-existing barrier dysfunction and necroinflammation in a crescendo of local intestinal necrosis and systemic inflammation/infection, as occasionally observed in patients on ion-exchange resin therapy.

## 1. Introduction

Oral intake of ion-exchange resins is clinically used to prevent certain dietary components from absorption in the intestinal tract. For example, the anion-exchange resins sevelamer and cholestyramine are used to trap phosphate and bile and the cation-exchange resins polystyrene sulfonate and patiromer are used to trap potassium. Although such ion-exchange resins are usually well tolerated, they are occasionally associated with life-threatening intestinal necrosis [1]. Such patients experience a sudden onset of abdominal pain, rectal bleeding, diarrhea, and hematochezia, which can progress to overt peritonitis, sepsis, and multiorgan failure. Risk factors for drug crystal-related intestinal necrosis include constipation, abnormal digestive secretion and absorption, intestinal hypoperfusion (e.g., during hemodialysis), surgery, immunosuppressive or non-steroidal anti-inflammatory drugs, colonic distension reducing blood flow, and chronic kidney disease (CKD) [2,3,4,5]. Obesity, diabetes mellitus, hypertension, and liver and heart failure also impair the intestinal barrier [6,7,8]. The role of ion-exchange resins in these complications is unknown.

All ion-exchange resins are organic polymers forming a porous insoluble matrix in the form of small microbeads (patiromer) or crystals (cholestyramine, sevelamer, polystyrene sulfonate). Upon oral intake, the fabricated product disintegrates in the upper intestinal tract to the micrometer scale, which maximizes the surface area available for ion exchange. By their dimension and shape, these ion-exchange resins resemble other drug crystals forming in the bile or urinary tract that are known to induce stones and epithelial barrier dysfunction and injury.

The last decade generated a lot of progress in the understanding of crystal biology and crystal-related disorders, referred to as crystallopathies [9]. Crystals induce inflammation by activating the NLRP3 inflammasome in resident macrophages and dendritic cells, followed by the release of interleukin (IL)-1β. Crystals also directly trigger various forms of regulated necrosis, e.g., necroptosis [10], ferroptosis [11], or mitochondrial permeability transition-related cell necrosis [12]. Of note, crystals may cause necrosis of neutrophils, together with the release of neutrophil extracellular traps (NETs) [13], a process contributing to many forms of sterile inflammation, e.g., in gout [14] or bile stone disease [15]. 

We therefore hypothesized that crystals of ion-exchange resins would trigger cell necrosis in intestinal epithelial cells as well as neutrophil necrosis and NET release as a mechanistic explanation for this complication of ion-exchange resin therapy.

## 2. Material and Methods

### 2.1. Clinical and Histological Evaluation of a Case with Sevelamer Crystal-Induced Colon Necrosis

We describe a case of sevelamer crystal-associated intestinal necrosis. A 67-year-old man was admitted with chronic kidney disease (CKD stage G4A3) and kidney atrophy to the Department of Nephrology at the Medical University of Graz in 2017. Paraffin-embedded sections from the colonic resection specimen were stained either with periodic acid Schiff (PAS) or hematoxylin and eosin (H&E) using standard protocols and analyzed by light microscopy. Representative images were captured to illustrate the extent of intestinal necrosis, sevelamer crystal deposits, and inflammatory cell infiltration.

To test for the presence of NETs [16], the colon biopsy section was stained with 4′,6-diamidin-2-phenylindol (DAPI, indicates cell nuclei; Sigma-Aldrich) and the neutrophil elastase antibody (Santa Cruz Biotechnology, Santa Cruz, CA, USA) for 1 h. After washing, the section was incubated with the secondary antibody rabbit AlexaFluor555 (Thermo Fisher Scientific, Planegg, Germany) for 30 min at room temperature. Fluorescence images were detected using a Leica TL light-emitting diode fluorescence microscope (Leica, Wetzlar, Germany). The study was approved by the local ethical department at the Medical University in Graz, Austria.

### 2.2. Isolation of Human Blood Neutrophils and CD14+ Monocytes

The study to obtain whole blood samples from healthy volunteers was approved by the local Ethical Review Board of the Medical Faculty at the Ludwig-Maximilians-University Hospital Munich (Ref.-No. 167-12). Informed consent was obtained from all subjects. Blood from human healthy individuals was collected in S-Monovette with lithium-heparin gel (Sarstedt, Numbrecht, Germany). An equal volume of 1.25% dextran solution was added and, after careful mixing, samples were stored at 4 °C for 20 min to allow the red blood cells to settle. The supernatant was washed with Dulbecco’s Phosphate-Buffered Saline (D-PBS) and centrifuged at 1300 rpm at 4 °C for 5 min. The pellet was lysed for 20 s using 10 mL of filtered H_2_O and the reaction was stopped by adding a 4 mL 0.6 M KCL solution. The solution was washed with D-PBS and centrifuged at 1300 rpm at 4 °C for 5 min. The pellet was resuspended in 4 mL of D-PBS and layered carefully on top of 4 mL Biocoll in a 15 mL Falcon tube. The tube was then centrifuged at 1500 rpm at 4 °C for 5 min without a break to separate the peripheral blood mononuclear cell layer from the neutrophil granulocyte pellet. The peripheral blood mononuclear cell layer was transferred into a new tube and the remaining supernatant was removed [17]. 

Human CD14+ monocytes were purified from the peripheral blood mononuclear cell layer by magnetic activated cell sorting with human CD14 microbeads for positive selection (Miltenyi Biotec, Bergisch Gladbach, Germany) [18], according to the manufacturer’s protocol.

Human CD14+ monocytes and neutrophils were suspended in serum-free Roswell Park Memorial Institute medium (Thermo Fisher Scientific, Planegg, Germany) and seeded either in 24-well plates, 96-well plates, or 8-well chambers in a 5% carbon dioxide atmosphere at 37 °C for 30 min depending on the desired analysis before stimulation with crystals.

### 2.3. Culture and Stimulation of Human Intestinal Epithelial Cells

Human Caco2 and HCA7 intestinal epithelial cell lines were obtained from ATCC or Sigma-Aldrich, Germany, respectively. Caco2 (2 × 10^4^ cells/well) and HCA7 (5 × 10^3^ cells/well) cells were cultured in Dulbecco’s modified Eagle’s medium (Merck, Darmstadt, Germany) containing 10% Fetal Bovine Serum (FBS) (Merck, Darmstadt, Germany) and 1% penicillin and streptomycin (PAN-BioTech, Aidenbach, Germany) in 96-well plates at 37 °C in a 5% carbon dioxide atmosphere. Sevelamer (Sanofi-Aventis, Frankfurt am Main, Germany), polystyrene sulfonate (MERCK, Darmstadt, Germany), cholestyramine (Ratiopharm, Ulm, Germany), patiromer (Vifor Fresenius Medical Care Renal Pharma, Paris La Défense CEDEX, France), and monosodium urate (Invivogen, San Diego, CA, USA) drugs/crystals were pestled and suspended in D-PBS prior to sonication to generate small crystals using an Ultrasonic Sonifier (Branson) for 5 min. After intestinal epithelial cells were 80% confluent, cells were stimulated with or without the aforementioned crystal types (200 or 1000 µg/mL) for 24 h. Supernatants were collected and stored at −20 °C until further analysis, and cells were harvested for flow cytometry or fluorescence microscopy.

### 2.4. Crystal Preparation and Ultrastructural Analysis of Crystals by Electron Microscopy

For scanning electron microscopy (SEM), sevelamer, polystyrene sulfonate, cholestyramine, patiromer, and monosodium urate crystals were mounted on a stub and coated with a thin layer of gold. The analysis was carried out on an FEI Quanta 250 (Hillsboro, OR, USA).

### 2.5. Lactate Dehydrogenase Cytotoxicity Assay

After stimulation of intestinal epithelial cells with crystals (see Section 2.3), supernatants from the cell culture were transferred to a new 96-well plate. To avoid noise from the autofluorescence of crystals, the supernatants were centrifuged and transferred to another 96-well plate without any excess crystals. The cytotoxicity was measured using the lactate dehydrogenase (LDH) cytotoxicity assay kit (Sigma-Aldrich, Darmstadt, Germany) according to the manufacturer’s protocol. The optical density was quantified via a 96-well plate reader to record the absorbance at 492 nm and the percentage of cytotoxicity was determined. Unstimulated cells were used as a negative control and 1% triton as a positive control.

### 2.6. Metabolic Activity Assay

The 3-(4,5-dimethylthiazol-2-yl)-2,5-diphenyltetrazolium bromide (MTT) assay was performed after stimulation of intestinal epithelial cells with or without crystals to evaluate their metabolic activity. The assay was conducted according to the manufacturer’s instructions. The optical density was quantified using a 96-well plate reader to record the absorbance at 570 nm and was normalized through untreated negative controls and positive controls treated with 1% triton. 

### 2.7. Visualization of Extracellular DNA Release by Confocal Microscopy

Human blood neutrophils and CD14+ monocytes were cultured in serum-free Roswell Park Memorial Institute (RPMI) medium in 8-well chamber slides (7.5 × 10^5^ cells/well, Nunc Lab-Tek, Sigma-Aldrich, Germany) and stimulated with various crystal types at different concentrations for 2 h [19]. After incubation, the culture medium was removed, the cells were fixed using 4% paraformaldehyde for 10 min at room temperature and washed twice with D-PBS, and the membrane was permeabilized using 0.1% triton in D-PBS for 10 min. The cells were then stained with phalloidin green dye for 40 min (165 nM, Sigma-Aldrich), washed with D-PBS, mounted with 4′,6-diamidin-2-phenylindol (DAPI, indicates cell nuclei; Sigma-Aldrich), and visualized using a Leica confocal microscope (UV and 488 nm) (Leica, Wetzlar, Germany). 

### 2.8. Cell Death Detection Enzyme Linked Immunosorbent Assay

Human blood neutrophils were cultured in serum-free RPMI medium in 96-well chamber slides (7.5 × 10^5^ cells/well, Nunc Lab-Tek, Sigma-Aldrich, Germany) and stimulated with various ion-exchange resin crystals (1000 µg/mL) for 2 h [19]. After incubation, supernatants were collected and transferred into a new 96-well plate. To remove any excess crystals from the supernatants, the plate was centrifuged and the supernatants transferred into another 96-well plate. Afterwards, supernatants were treated with or without DNAse to remove DNA from the supernatant (as a control). The release of histone-complexed DNA fragments from NETs was quantified using the cell death detection enzyme linked immunosorbent assay (ELISA)^PLUS^ (MERCK, Darmstadt, Germany) according to the manufacturer’s protocol. 

### 2.9. Flow Cytometry Analysis

To characterize human Caco2 and HCA7 intestinal epithelial cells by flow cytometry, both cell lines were stained with the surface antibodies FITC anti-human CD14 (BioLegend, Fell, Germany), and AlexaFluor488 anti-pan cytokeratin or AlexaFluor488 anti-mouse IgG1κ isotype control (both from Invitrogen, Carlsbad, CA, USA). The mean fluorescence intensity (MFI) was quantified and compared with the expression levels of human blood neutrophils and CD14+ monocytes.

To evaluate cell death, human intestinal epithelial cells were cultured with or without various crystal types (200 or 1000 µg/mL) in complete DMEM for 24 h. Human blood neutrophils were stimulated with or without sevelamer, polystyrene sulfonate, and cholestyramine (1000 µg/mL) in serum-free RPMI medium for 2 h. Neutrophils were also stimulated with monosodium urate crystals in the absence or presence of the necrosis inhibitor necrostatin-1s (Nec1s, 30 µM) for 2 h, which served as a positive control. After stimulation, intestinal epithelial cells and neutrophils were stained using the AnnexinV-FITC detection kit with propidium iodide (PI) (BioLegend, Fell, Germany) to distinguish among live (AnnexinV-PI-), apoptotic (AnnexinV+PI-), late apoptotic/primary necrotic (AnnexinV+PI+) and necrotic (AnnexinV-PI+) cells. Flow cytometry analysis was performed on a FACSCalibur (BD Biosciences, Heidelberg, Germany), and data were analyzed using the software FlowJo 8.7 (Tree Star, Ashland, OR, USA). 

To determine the release of myeloperoxidase (MPO) by human neutrophils upon stimulation with different ion-exchange resin crystals, supernatants were collected after 2 h and stained with the FITC anti-human MPO or the FITC mouse IgG1 isotype control (BioLegend, Fell, Germany) for 15 min. Flow cytometry analysis was performed on a FACSCalibur (BD Biosciences, Heidelberg, Germany) and data were analyzed using the software FlowJo 8.7 (Tree Star, Ashland, OR, USA). 

### 2.10. Electric Cell-Substrate Impedance Sensing

A total of 30,000 Caco2 intestinal epithelial cells/well were seeded into electric cell-substrate impedance sensing (ECIS) array (8W10Eþ) chambers (ibidi, Martinsried, Germany) with Dulbecco’s modified Eagle’s medium containing 10% FBS [20,21]. Data analysis was performed as described by Wegener et al. with slight modifications (Figure 4A,B) [20,21]. In brief, the confluent condition of Caco2 cells was evaluated through stable capacitance. After confluence of cells was reached after 22 h, the medium was changed to FBS-free medium, and 1000 µg/mL sevelamer or polystyrene sulfonate crystals or the medium alone were added in each well. Monosodium urate crystals (1000 µg/mL) were used as a positive control. The capacitance was monitored and recorded for up to 65 h. HCA7 cells grow in multiple cell layers; therefore, they were not considered for such experiments.

### 2.11. Statistical Analysis

Statistical analysis was performed using GraphPad Prism 7.0 software (GraphPad, San Diego, CA, USA). Data were compared either by one-way ANOVA with Tukey’s post-hoc test to calculate significance among three or more groups; two-way ANOVA with Bonferroni’s comparison post-hoc test was carried out when using two parameters with multiple groups. Data are presented as means ± SD. Differences were considered significant if *p* ≤ 0.05; ns indicates not significant (*p* > 0.05). Sample sizes are indicated in each corresponding figure legend.

## 3. Results

### 3.1. Sevelamer Crystal-Related Intestinal Necrosis Involves Local Formation of Neutrophil Extracellular Traps

**Figure 1:**
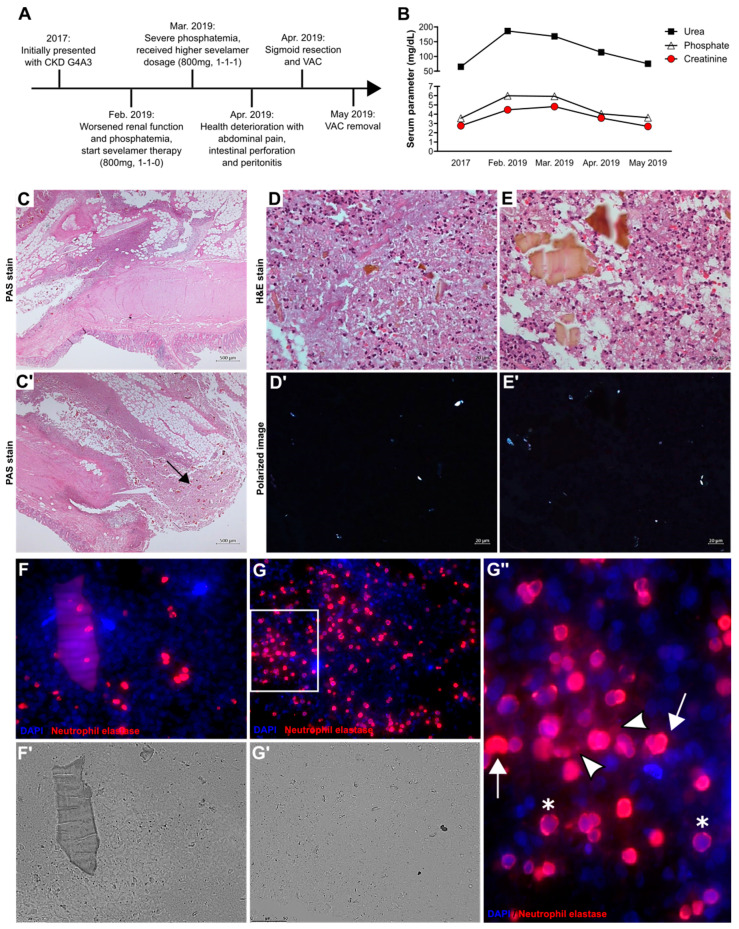
Sevelamer crystal-induced intestinal necrosis associated with neutrophil extracellular trap formation. (**A**) Clinical course of a patient (67 years of age) with chronic kidney disease (CKD). The intake of sevelamer due to phosphatemia and the prolonged decline in kidney function caused intestinal necrosis, perforation, and peritonitis. Surgery with vacuum-assisted closure (VAC) was needed and the patient recovered. Sevelamer therapy was discontinued. (**B**) Serum urea, phosphate, and creatinine levels over the clinical course of the same patient. (**C**,**C’**) Periodic acid-Schiff (PAS) staining of sections of the colonic resection specimen from the patient. The arrow indicates sevelamer crystal deposits. Magnification 25×. (**D**,**E**) Hematoxylin and eosin (H&E) staining of the same resection specimen showing necrotic lesions, infiltration of cells, fibrosis, and massive sevelamer crystal deposits. Only small sevelamer crystals are birefringent under polarized light but not big crystal masses (**D’**,**E’**). (**F**,**F’**,**G**,**G’**) Fluorescence (**F**,**G**) and light (**F’**,**G’**) microscopy of the same colonic resection specimen stained with 4′,6-diamidin-2-phenylindol (DAPI) (nuclei/DNA, blue) and neutrophil elastase to identify neutrophil extracellular trap (NET)-like structures, red. (**G’’**) A close-up representative image indicating decondensed non-aggregated NETs (white arrowheads), diffuse NETs with decondensed nuclei (DNA) (white arrows), and neutrophils with intact DNA/nuclei (*).

A 67-year-old man with chronic kidney disease stage G4A3 and kidney atrophy was admitted to the Department of Nephrology at the Medical University of Graz in 2017. His past medical history included chronic hyponatremia, hypertension, schizophrenic paranoia, syncope, renal anemia, and chronic nicotine abuse. In February 2019, he returned to the hospital due to deterioration of kidney function and hyperphosphatemia (Figure 1A). Serum creatinine, urea, and phosphate levels had increased compared with the first visit in 2017 (Figure 1B). Therapy with the phosphate binder sevelamer (800 mg two times per day) was started. One month later, no improvement of phosphate levels had occurred (Figure 1B); therefore, the dose of sevelamer was increased to 800 mg three times per day. Mid-April 2019, he was readmitted to the hospital with abdominal pain. The patient underwent right hemicolectomy. Macroscopic and histological examination of the surgical specimen revealed colon perforation, confluent fibrinoid necrosis, and ulceration of the colonic mucosa with diffuse peritonitis and sevelamer crystals (black arrow) (Figure 1C,C’). Sevelamer crystals were displayed as irregularly spaced “fish scales” of different sizes (Figure 1D,E, top panel). Smaller sevelamer crystals were birefringent under polarized light (Figure 1D’,E’, bottom panel). The dilated sigmoid colon was resected with a transition point, an end colostomy with Hartmann’s pouch was created, and vacuum-assisted closure therapy was initiated. Sevelamer medication was discontinued. After four weeks, the vacuum-assisted closure was removed and the patient could be discharged from the hospital. Despite this severe complication, no dialysis was required. 

Since crystalline particles can trigger neutrophil necrosis and NET release [13], we investigated NETs in the tissue samples of this case of sevelamer crystal-related intestinal necrosis. Immunofluorescence and light microscopy of the colon biopsy showed that neutrophil elastase-positive neutrophils (red color) did not localize around large sevelamer crystals (Figure 1F,F’) but instead in areas with small crystals (Figure 1G,G’). At a higher magnification, abundant decondensed non-aggregated NETs (white arrowheads), diffuse NETs with decondensed nuclei (white arrows), and neutrophils with intact nuclei (*) were observed (Figure 1G’’). Thus, sevelamer therapy-related intestinal necrosis is associated with NET formation in proximity to small sevelamer crystals.

### 3.2. Crystals of Ion-Exchange Resins Reduce the Metabolic Activity of Intestinal Epithelial Cells

**Figure 2:**
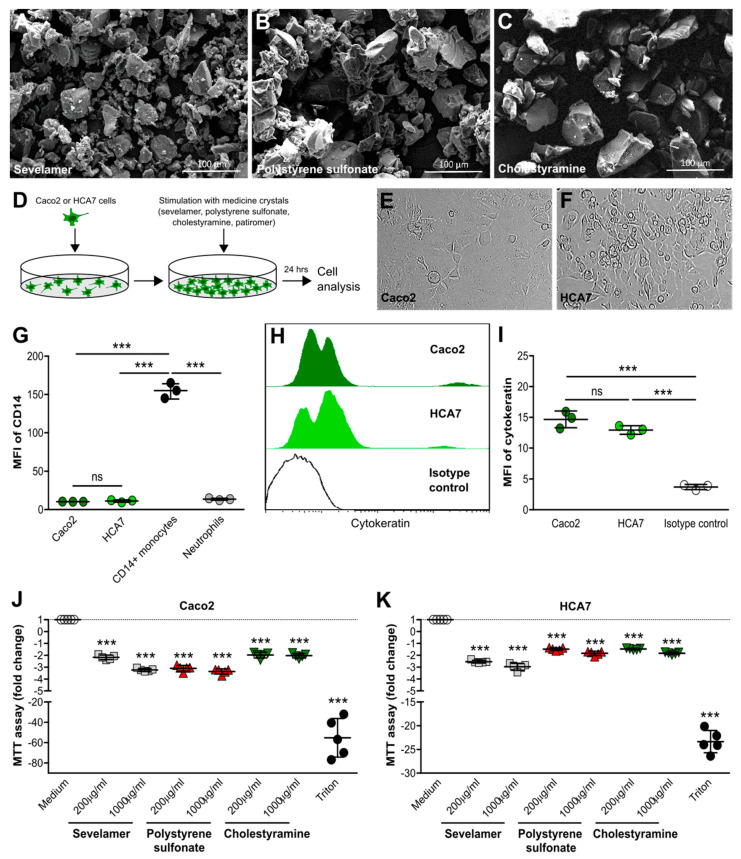
Crystals of ion-exchange resins reduce metabolic activity in intestinal epithelial cells. (**A**–**C**) Scanning electron microscopy illustrates sevelamer (**A**), polystyrene sulfonate (**B**), and cholestyramine (**C**) crystals of different sizes and shapes. (**D**) Schematic of in vitro culture set-up of human Caco2 and HCA7 intestinal epithelial cell lines. (**E**,**F**) Light microscopy of human Caco2 (**E**) and HCA7 (**F**) intestinal epithelial cell lines (400× magnification). (**G**) Caco2 and HCA7 cells, as well as human blood neutrophils and CD14+ monocytes, were stained for the surface marker CD14 and analyzed by flow cytometry (*n* = 3). (**H**,**I**) Caco2 and HCA7 cells with cytokeratin or isotype control (using CaCo2 cells) and flow cytometry performed. Histogram (**H**) and the mean fluorescence intensity (MFI) (**I**) are shown (*n* = 3). (**J**,**K**) Human Caco2 and HCA7 intestinal epithelial cell lines were stimulated with different concentrations of sevelamer, polystyrene sulfonate, and cholestyramine crystals (200 or 1000 µg/mL) for 24 h. Triton served as a positive control. After stimulation, culture supernatants were collected and 3-(4,5-dimethylthiazol-2-yl)-2,5-diphenyltetrazolium bromide (MTT) assays were performed. The fold change is shown (*n* = 5). Data are means ± SD. * *p* ≤ 0.05; ** *p* ≤ 0.01; *** *p* ≤ 0.001; ns, not significant (*p* > 0.05) versus the medium using one-way ANOVA.

To investigate the effects of crystals of various ion-exchange resins on intestinal epithelial cells, we used sevelamer, polystyrene sulfonate, and cholestyramine crystals that were similar in size and shape (Figure 2A–C) to the small round-shaped patiromer beads and needle-like monosodium urate crystals (Figure S1A,B) as captured by scanning electron microscopy. In vitro (Figure 2D), human Caco2 intestinal epithelial cells formed a uniform monolayer (Figure 2E), whereas human HCA7 intestinal epithelial cells grew in several cell layers (Figure 2F). Flow cytometry revealed that both Caco2 and HCA2 cells, similar to human blood neutrophils, did not express the surface marker CD14 compared with human blood CD14+ monocytes, as indicated by the histogram and mean fluorescence intensity (Figure 2G), whereas both cell lines expressed the epithelial marker cytokeratin (Figure 2H,I). Stimulation of Caco2 cells with different concentrations of sevelamer, polystyrene sulfonate, and cholestyramine crystals (Figure 2J), as well as patiromer and monosodium urate crystals (Figure S1C), resulted in a significant decrease in metabolic activity compared with the medium control after 24 h. We observed similar results with HCA7 cells (Figure 1K and Figure S1D). Thus, crystals of ion-exchange resins diminish the metabolic activity of intestinal epithelial cells.

### 3.3. Crystals of Ion-Exchange Resins Induce Cell Death in Intestinal Epithelial Cells

**Figure 3:**
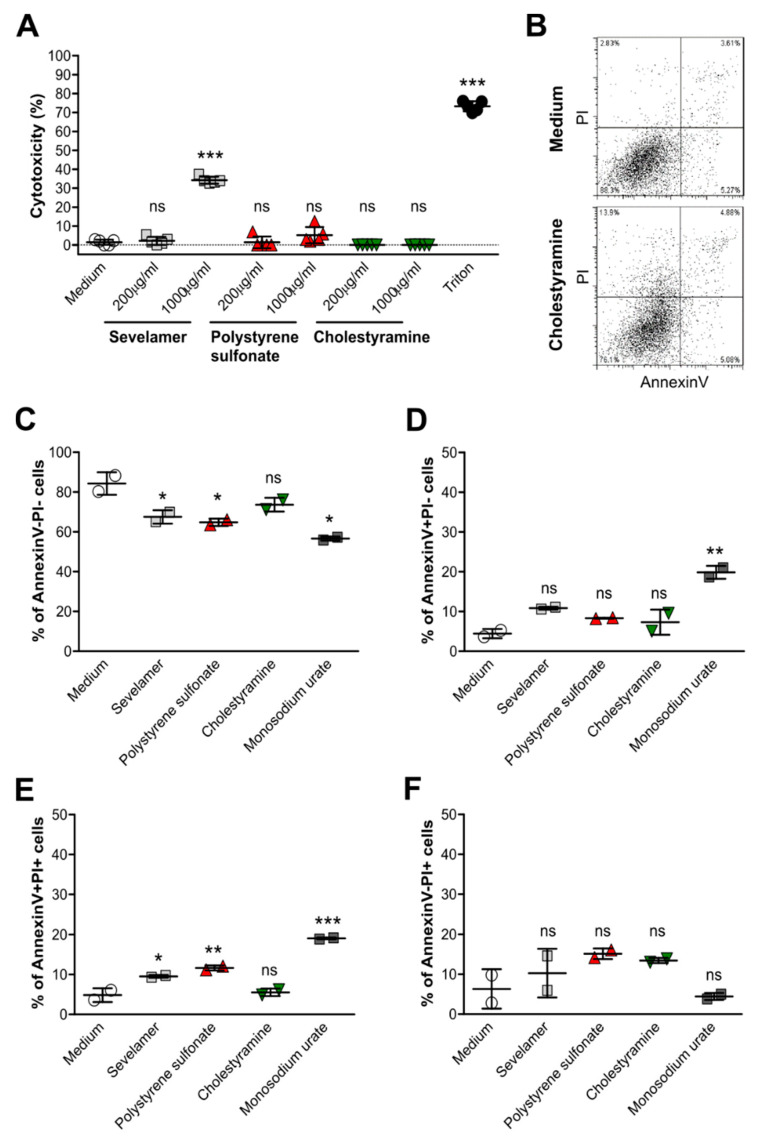
Crystals of ion-exchange resins induce cell death in Caco2 intestinal epithelial cells. Human Caco2 intestinal epithelial cell lines were stimulated with different concentrations (200 or 1000 µg/mL) of sevelamer, polystyrene sulfonate, cholestyramine, or monosodium urate crystals for 24 h. Triton was used as a positive control. (**A**) After stimulation, culture supernatants were collected and lactate dehydrogenase (LDH) assays was performed. Cytotoxicity is presented as a percentage (%) (*n* = 5). (**B**–**F**) AnnexinV/propidium iodide (PI) staining of Caco2 cells was performed by flow cytometry (**B**). The percentage of live (AnnexinV-PI-) (**C**), apoptotic (AnnexinV+PI-) (**D**), late apoptotic/early necrotic (AnnexinV+PI+) (**E**), and necrotic (AnnexinV-PI+) (**F**) Caco2 cells after stimulation with 1000 µg/mL of different drug crystals was determined (*n* = 2). Data are means ± SD. * *p* ≤ 0.05; ** *p* ≤ 0.01; *** *p* ≤ 0.001; ns, not significant (*p* > 0.05) versus the medium using one-way ANOVA.

To investigate whether crystals of ion-exchange resins can cause cell death in intestinal epithelial cells and therefore contribute to the intestinal complications observed in patients [22,23,24,25], including our case, we stimulated Caco2 and HCA7 cells with different concentrations of sevelamer, polystyrene sulfonate, cholestyramine, patiromer, and monosodium urate crystals as a control for 24 h, and performed cytotoxicity assays. Only a very high concentration of sevelamer crystals induced a significant increase in the percentage of cytotoxicity in Caco2 cells compared with the medium and other drug crystals (Figure 3A). This was also the case for patiromer and monosodium urate crystals (Figure S1E). On the other hand, such crystals had no cytotoxic effect on HCA7 cells unlike monosodium urate crystals (Figures S2A and S1F).

Next, to evaluate the effects of crystals of ion-exchange resins on epithelial cell death in more detail, we stained resin crystal-stimulated intestinal epithelial cells with AnnexinV and propidium iodine (PI) (Figure 3B). Flow cytometry revealed that the number of live (AnnexinV-PI-) (Figure 3C) cells decreased upon crystal stimulation compared with the medium, whereas the number of apoptotic (AnnexinV+PI-) (Figure 3D), late apoptotic/early necrotic (AnnexinV+PI+) (Figure 3E), and necrotic (AnnexinV-PI+) (Figure 3F) cells increased in Caco2 cells, although this was not always significant. Drug crystals induced only an increase in the number of necrotic HCA7 cells (AnnexinV-PI+) (Figure S2F) but not apoptotic and late apoptotic/early necrotic cells (Figure S2D,E) compared with the medium (Figure S2C–F). Taken together, crystals of ion-exchange resins can lead to cell death in intestinal epithelial cells. 

### 3.4. Crystals of Ion-Exchange Resins Induce Intestinal Epithelial Barrier Dysfunction

**Figure 4:**
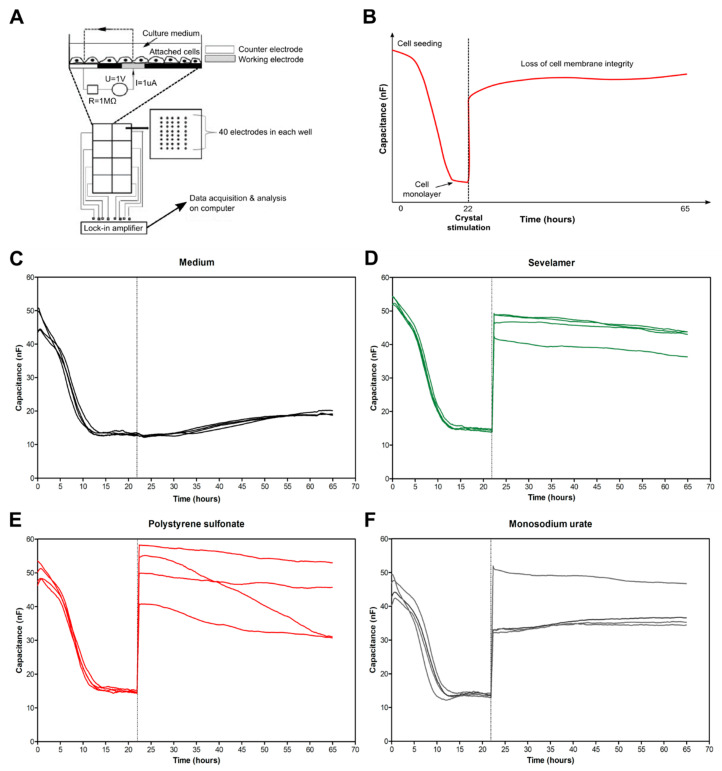
Crystals of ion-exchange resins induce intestinal epithelial barrier dysfunction. (**A**) Schematic of the electric cell-substrate impedance sensing (ECIS) working principle. Caco2 cells (30,000 cells/well) were grown on electrodes and covered in culture medium. The electrodes were connected to a lock-in amplifier and an AC signal was applied via a 1MX resistor to create a constant current. There are 40 electrodes in each well to measure the average capacitance in approximately 2000–4000 cells. Capacitance was applied at 16,000 Hz. (**B**) Schematic of the ECIS analysis. Cells were seeded on electrodes and when they were confluent after 22 h, the capacitance became stable. Afterwards, the medium was changed and the cells were stimulated with or without different crystal types for up to 65 h. Data are presented as normalized capacitance. (**C**–**F**) Images show the capacitance (nF) of Caco2 cells cultured in the medium alone (**C**), with sevelamer (1000 µg/mL) (**D**), polystyrene sulfonate (1000 µg/mL) (**E**), or monosodium urate (1000 µg/mL, positive control) (**F**) crystals (*n* = 4).

Advanced CKD can be associated with intestinal barrier dysfunction [7,26,27]. It is unknown whether crystals of phosphate and potassium binders that are frequently used in CKD contribute to this phenomenon. Therefore, we performed electric cell-substrate impedance sensing as previously described [21]. This allowed us to monitor intestinal epithelial monolayer injury online in the absence or presence of sevelamer and polystyrene sulfonate crystals or monosodium urate crystals as a control. Schematic diagrams illustrating the experimental design are shown in Figure 4A,B. Human Caco2 cells were seeded in wells onto electrodes until they formed a cell monolayer and reached 80% confluence, as illustrated by a low normalized capacitance (Figure 4B). After 22 h, fresh medium was added and Caco2 cells were stimulated with or without different drug crystals at high concentrations (1000 µg/mL) for up to 65 h. Compared with the medium (Figure 4C), sevelamer (Figure 4D) and polystyrene sulfonate (Figure 4E) crystals, as well as monosodium urate crystals (Figure 4F), impaired the integrity of the Caco2 cell monolayer, as indicated by an increase in the capacitance. Our data suggest that anion- and cation-exchange drug crystals also cause barrier dysfunction in intestinal epithelial cells.

### 3.5. Crystals of Ion-Exchange Resins Induce Neutrophil Extracellular Trap Formation

**Figure 5:**
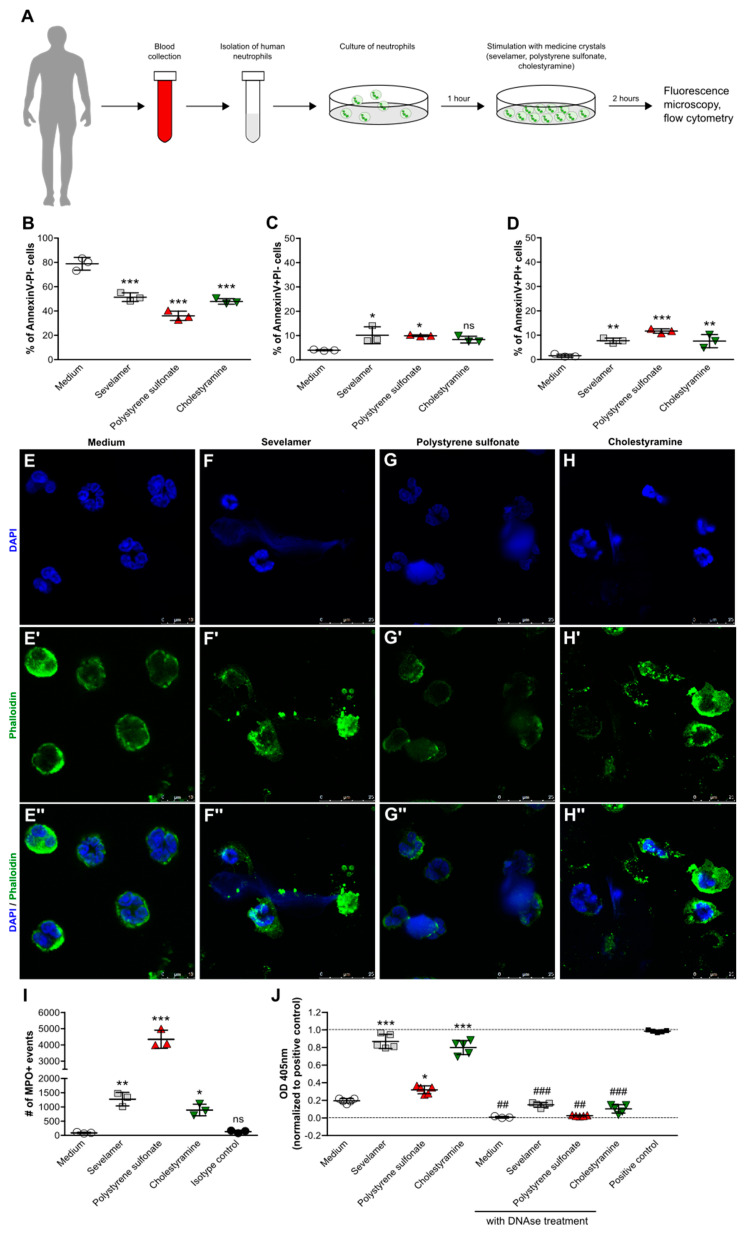
Crystals of ion-exchange resins induce neutrophil extracellular trap formation. (**A**) Schematic showing work flow. Human blood neutrophils were isolated from healthy individuals and stimulated with or without sevelamer, polystyrene sulfonate, or cholestyramine crystals (1000 µg/mL) for 2 h. (**B**–**D**) After stimulation, the percentage of live (AnnexinV-PI-) (**B**), apoptotic (AnnexinV+PI-)(**C**), and late apoptotic/necrotic (AnnexinV+PI+) (**D**) neutrophils was quantified by flow cytometry (*n* = 3 from 3 donors). (**E**–**H**) Neutrophils were stained with DAPI (blue, stains nuclei) (**E**–**H**) and phalloidin (green, stains actin) (**E’**–**H’**) to visualize neutrophil extracellular trap formation in response to sevelamer (**F**), polystyrene sulfonate (**G**), and cholestyramine (**H**) crystals or the medium control (**E**). Images are also shown as a merge of DAPI and phalloidin (400× or 630× magnification (**E’’**–**H’’**)). (**I**) The number of myeloperoxidase (MPO)-positive events was determined in the supernatants of human neutrophils stimulated with or without sevelamer, polystyrene sulfonate, or cholestyramine crystals (1000 µg/mL) after 2 h by flow cytometry. (**J**) Quantification of released histone-complexed DNA fragments (OD 450 nm) in culture supernatants of crystal-stimulated neutrophils after 2 h using a cell death detection ELISA^PLUS^ assay. DNAse treatment was used as the control. Data are means ± SD. * *p* ≤ 0.05; ** *p* ≤ 0.01; *** *p* ≤ 0.001; ns, not significant (*p* > 0.05) versus the medium, and ## *p* ≤ 0.01; ### *p* ≤ 0.001 versus without DNAse treatment, using one-way ANOVA.

To confirm that ion-exchange resin crystals can directly trigger cell death in human neutrophils, we isolated human blood neutrophils from healthy individuals and stimulated them with or without crystals for 2 h (Figure 5A). Flow cytometry for AnnexinV/PI, a cytotoxicity assay, and fluorescence microscopy using the nuclear marker DAPI and the actin stain phalloidin were performed. Flow cytometry analysis revealed that in the presence of sevelamer, polystyrene sulfonate, and cholestyramine crystals, the percentage of live (AnnexinV-PI-) neutrophils significantly decreased (Figure 5B), whereas the number of AnnexinV+PI-) (Figure 5C) and AnnexinV+PI+ (Figure 5D) neutrophils significantly increased. This was in line with our data showing that monosodium urate crystals induced cytotoxicity in human neutrophils (Figure S3A) and increased the percentage of AnnexinV+PI+ cells (Figure S3B). This process could be partially inhibited by the necroptotis inhibitor necrostatin-1s (Figure S3A), consistent with AnnexinV surface positivity being not necessarily a marker of apoptosis but also of necroptosis [28]. Furthermore, like monosodium urate crystals (Figure S3C–C’’), ion-exchange resin crystals triggered chromatin decondensation and diffuse NET release as illustrated by the NET-like extracellular DNA (DAPI, blue) and membrane rupture (phalloidin, green) (Figure 5F–H) compared with the medium (Figure 5E). This process was associated with the release of MPO (Figure 5I) and histone-complexed DNA fragments (Figure 5J) by neutrophils in the presence of drug crystals as well as monosodium urate crystals (Figure S3D,E). Thus, crystalline ion-exchange resins induce direct cell death in neutrophils, which is associated with the release of NET-like structures.

### 3.6. Crystals of Ion-Exchange Resins Induce Neutrophil Extracellular Trap Formation

**Figure 6:**
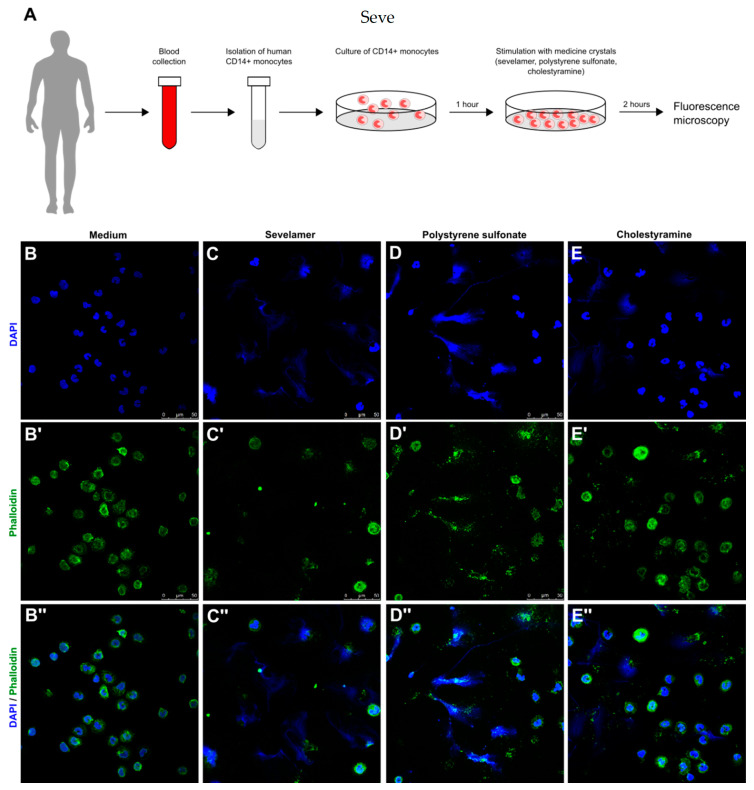
Crystals of ion-exchange resins induce extracellular trap formation in human CD14+ monocytes. (**A**) Schematic showing work flow. Human blood CD14+ monocytes were isolated from healthy individuals and stimulated with or without sevelamer, polystyrene sulfonate, or cholestyramine crystals (1000 µg/mL) for 2 h. (**B**–**E**) After stimulation, CD14+ monocytes were stained with DAPI (blue, stains nuclei) (**B**–**E**) and phalloidin (green, stains actin) (**B’**–**E’**) to visualize extracellular trap formation in response to sevelamer (**C**), polystyrene sulfonate (**D**), and cholestyramine (**E**) crystals or the medium (control) (**B**). Images are also shown as a merge of DAPI and phalloidin (200× magnification **(B’’**–**E’’**)).

Human monocytes and macrophages can also release chromatin in a NET-like manner, referred to as monocyte/macrophage extracellular traps (METs) [29,30]. Whether drug crystals trigger this process is unknown. Therefore, we isolated blood CD14+ monocytes from healthy individuals and exposed them to crystals of sevelamer, polystyrene sulfonate, and cholestyramine for 2 h (Figure 6A). Fluorescence microscopy using the nuclear marker DAPI (blue) and the actin stain phalloidin (green) showed that crystals from all three ion-exchange resins caused membrane rupture and the release of extracellular DNA (Figure 6D–E) compared with the medium (Figure 6B). Thus, crystals of ion-exchange resins induce MET-like structures similar to NETs.

### 3.7. Crystals of Ion-Exchange Resin-Induced Neutrophil Extracellular Traps Cause Cell Death in Intestinal Epithelial Cells 

**Figure 7:**
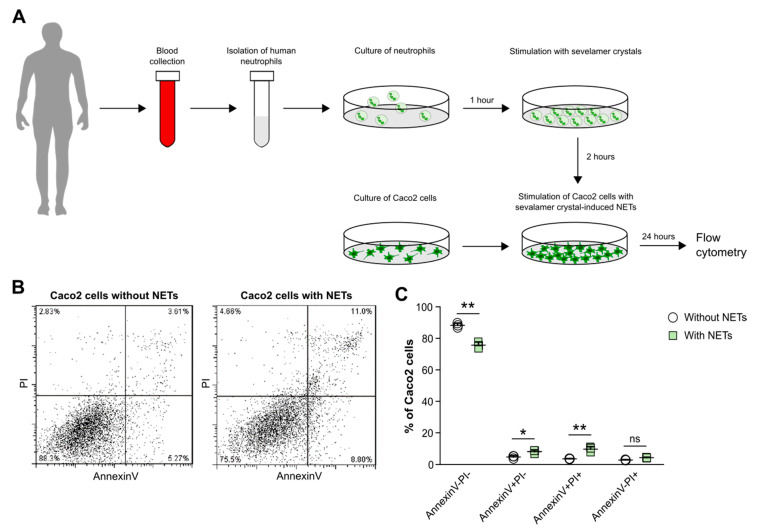
Soluble mediators released from ion-exchange resin crystal-induced neutrophil extracellular traps drive cell death in intestinal epithelial cells. (**A**) Schematic showing work flow. Human blood neutrophils were isolated from healthy individuals and stimulated with or without sevelamer crystals (1000 µg/mL). After 2 h, supernatants with or without neutrophil extracellular traps (NETs) were collected and incubated with Caco2 cells for 24 h. (**B**,**C**) After stimulation, the percentage of live (AnnexinV-PI), apoptotic (AnnexinV+PI-), and late apoptotic/necrotic (AnnexinV+PI+) neutrophils was quantified by flow cytometry (*n* = 3). Data are means ± SD. * *p* ≤ 0.05; ** *p* ≤ 0.01; ns, not significant (*p* > 0.05) using two-way ANOVA.

Finally, to investigate whether sevelamer crystal-induced NETs can indirectly trigger cell death in intestinal epithelial cells, we isolated blood neutrophils from healthy individuals and stimulated them with sevelamer crystals to induce NETs for 2 h. Afterwards, supernatants containing NET-like constituents including histone-complexed DNA fragments and MPO (Figure 5) were collected and added to Caco2 cells for 24 h (Figure 7A). Flow cytometry revealed that in the presence of NETs, the percentage of AnnexinV-PI- Caco2 cells decreased significantly compared with Caco2 cells cultured without NETs (Figure 7B,C). In contrast, the percentage of AnnexinV+PI- and AnnexinV+PI+ Caco2 cells significantly increased (Figure 7B,C). However, we did not observe a significant difference in the percentage of necrotic (AnnexinV-PI+) cells between Caco2 cells cultured with or without NETs. Taken together, our data indicate that sevelamer crystal-induced NETs promote cell death in intestinal epithelial cells and, therefore, may contribute to epithelial barrier dysfunction and intestinal injury.

## 4. Discussion

**Figure 8:**
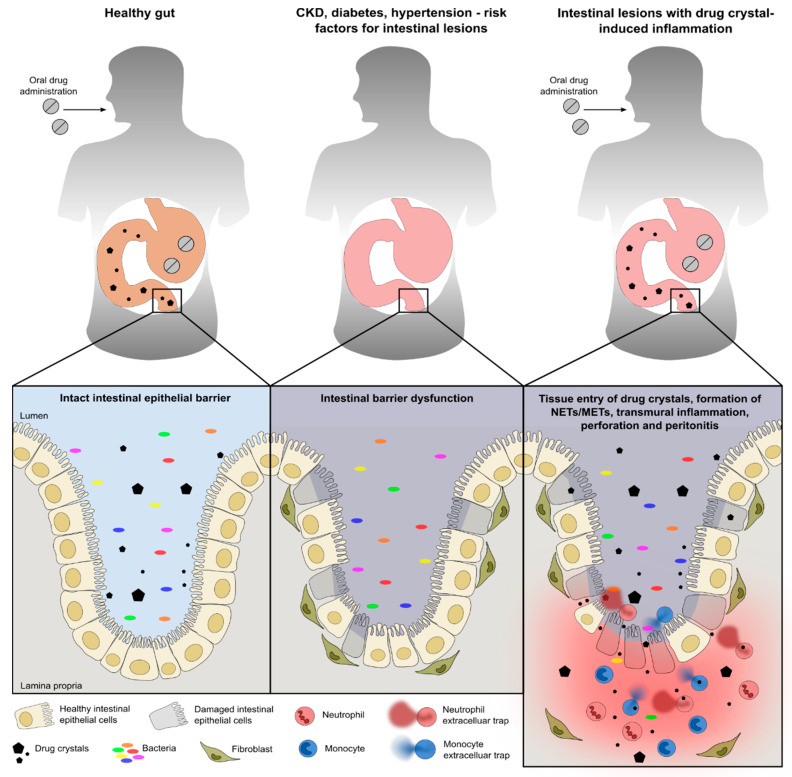
Schematic illustrating the pathogenesis of the intestinal epithelium during health and disease. Under healthy conditions, intestinal epithelial cells form a physical barrier that maintains segregation between the lumen and lamina propria/mucosa even after the intake of drugs (left panel). Chronic diseases such as hypertension, chronic kidney disease (CKD), diabetes, etc., can contribute to intestinal barrier dysfunction associated with intestinal epithelial cell death, leakage, fibrosis, and impaired epithelium regeneration (middle panel). This predisposes patients, in relation to the intake of drugs such as sevelamer, polystyrene sulfonate, and cholestyramine, to gastrointestinal injury and inflammation. Under certain conditions, drugs that are not completely digested and remain as crystalline particles in the intestine may further aggravate the intestinal barrier dysfunction that is associated with cell death, inflammation, fibrosis, and neutrophil and monocyte extracellular trap (NETs and METs) formation (right panel).

We hypothesized that crystals of sevelamer, polystyrene sulfonate, and cholestyramine can trigger the formation of NETs and METs, a process that contributes to intestinal barrier dysfunction and necrosis. Indeed, our data demonstrate that crystals of sevelamer, polystyrene sulfonate, and cholestyramine induce barrier dysfunction and cell death in human Caco2 and HCA7 intestinal epithelial cells. On the other hand, crystals of ion-exchange resins trigger the formation of human NETs and METs. Thus, the chronic inflammation associated with extracellular trap formation further drives intestinal necrosis upon contact with ion-exchange resin crystals (Figure 8). 

Intestinal necrosis induced by drug crystals is underrecognized and can pose a diagnostic challenge in patients with multiple chronic diseases. Sevelamer, cholestyramine, and polystyrene sulfonate are all ion-exchange resins that form crystalline fragments upon intestinal disintegration of the resin formulation [1,5,23,31]. Swanson et al. reviewed 15 cases of drug crystal fragments identified in intestinal biopsy specimens from four different academic centers [2]. The presence of sevelamer crystals was considered an incidental finding in some cases, while in others a direct association was suspected, given the findings of necrosis, ulceration, and crypt distortion [2]. The comorbidities of these patients with CKD included hypertension, diabetes mellitus, peripheral vascular disease, ulcerative disease, cholangitis, and human immunodeficiency virus. Our case of sevelamer crystal-related intestinal necrosis shared some of these risk factors and revealed numerous large and small sevelamer crystals inside the ulcerated intestinal wall, together with inflammation, NET formation, and tissue necrosis. In addition, crystals associated with cholestyramine and polystyrene sulfonate intake have also been found together with epithelial erosion, ulceration, and acute inflammation, predominantly in the colon [4,22,23,32,33,34]. 

Aberrant crystallization of organic materials such as calcium oxalate, monosodium urate, and cholesterol within the human body or exposure to external crystalline materials such as silica, asbestos, and air pollutants can cause inflammatory responses associated with pathogenesis in various acute and chronic diseases [9,35]. Whether intestinal crystal deposition of ion-exchange resins is an innocent bystander phenomenon or directly contributes to intestinal injury and necrosis is not well understood. Our in vitro data demonstrate that crystals of sevelamer, polystyrene sulfonate, and cholestyramine can directly induce barrier dysfunction in human Caco2 and HCA7 intestinal epithelial cells, while creating cytotoxic effects when crystals were used at a high concentration. The strong cytotoxicity of crystals, for example, has been reported in an animal model of acute kidney injury, whereby calcium oxalate crystals induce tubular epithelial cell necrosis [10,36]. This suggests that the barrier dysfunction caused by crystals of ion-exchange resins may contribute to pre-existing intestinal barrier dysfunctions in patients with multiple chronic diseases [6,7,37,38,39,40].

Several other mechanisms of crystal-induced inflammation have been proposed, including NET formation, a form of neutrophil death known as NETosis [41,42,43,44]. Indeed, crystals of sevelamer, polystyrene sulfonate, and cholestyramine can also trigger extracellular trap formation in human neutrophils and CD14+ monocytes, which we confirmed on a colonic resection specimen from a patient with sevelamer crystal-induced intestinal necrosis. Such extracellular traps release numerous pro-inflammatory proteases, damage-associated molecular patterns, alarmins, and cytotoxic histones [16,42,43,44,45], which, in turn, can elicit local intestinal inflammation and may indirectly drive intestinal necrosis, a process previously described as necroinflammation [46,47]. 

The limitations of our study are that we used human intestinal epithelial cell lines to investigate the impact of ion-exchange resin crystals on barrier dysfunction and immune cell function in vitro. It is possible that primary intestinal epithelial cells as well as immune cells from patients with multiple chronic medical comorbidities may respond differently compared with cell lines. Furthermore, all ion-exchange resins used in this study were mechanically disintegrated and sonicated to generate small crystals for in vitro stimulation. However, in humans, bacteria and metabolizing enzymes (e.g., monoamine oxidase and dopa decarboxylase) in the stomach and intestinal tract are responsible for digesting orally applied drugs. The digestive process and drug absorption might be altered in patients with preexisting chronic comorbidities [48,49]. Therefore, ion-exchange resin crystals may vary in size and shape and metabolizing enzymes could coat these crystals, which, in turn, may affect the function of intestinal epithelial cells and immune cells differently compared with healthy subjects. Finally, in vivo, any ulceration of the intestinal wall will promote the entry and translocation of microbes of the intestinal microbiota (Figure 8), which will certainly act as potent triggers for the local release of NETs and METs. To what extent drug crystals contribute as direct triggers to the overall inflammatory response and tissue destruction remains unclear at this point.

## 5. Conclusions

In conclusion, we found that crystals of sevelamer, polystyrene sulfonate, and cholestyramine induce intestinal epithelial cell barrier dysfunction as well as triggering the formation of NETs and METs. In addition, we confirmed NET formation in a CKD patient with sevelamer crystal-related intestinal necrosis. These data raise the possibility that under certain conditions, drug crystals may further contribute to the autoamplification of a pre-existing barrier dysfunction and necroinflammation in a crescendo of local intestinal necrosis and systemic inflammation/infection (Figure 8), as occasionally observed in CKD patients on ion-exchange resin therapy.

## Supplementary Materials

The following are available online at https://www.mdpi.com/2073-4409/9/11/2481/s1, Figure S1: Patiromer crystals reduce the metabolic activity in intestinal epithelial cells. Figure S2: Drug crystals induce only slight cell death in HCA7 intestinal epithelial cells. Figure S3: Monosodium urate crystals induce neutrophil extracellular trap formation.
