# Supplementary material for: Drug Crystal-Related Gastrointestinal Complications Involve Crystal-Induced Release of Neutrophil and Monocyte Extracellular Traps
Source: Cells. 2020 Nov 15;9(11):2481. doi: 10.3390/cells9112481 (PMC7697008; doi:10.3390/cells9112481)
Supplement: Supplementary file 1 [file cells-09-02481-s001.pdf]

cells

Supplementary Materials

Drug crystal-related gastrointestinal complications involve crystal-induced release of neutrophil and monocyte extracellular traps

Tehyung Kim [1], Sueli de Oliveira Silva Lautenschlager [2], Qiuyue Ma [1], Kathrin Eller [3], Marion Pollheimer [4], Danielle Lazarin-Bidóia [2], Celso Vataru Nakamura [2], Hans-Joachim Anders [1,*] and Stefanie Steiger [1,*]

Supplementary Materials: The following are available online at www.mdpi.com/xxx:
Figure S1: Patiromer crystals reduce the metabolic activity in intestinal epithelial cells.
Figure S2: Drug crystals induce only slight cell death in HCA7 intestinal epithelial cells.
Figure S3: Monosodium urate crystals induce neutrophil extracellular trap formation.

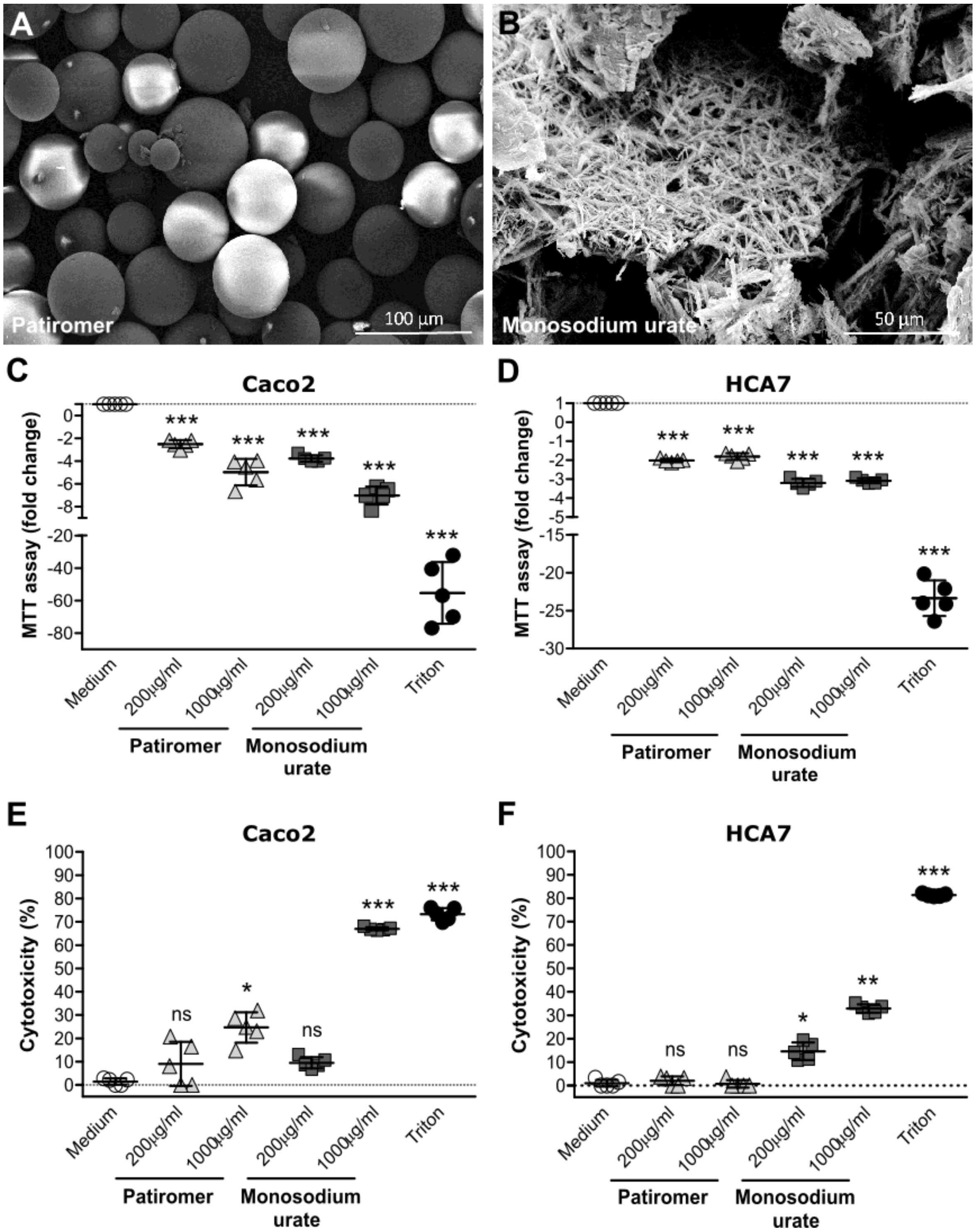

Figure S1: Patiromer crystals reduce the metabolic activity of intestinal epithelial ce**lls.** (**A**) Scanning electron microscopy illustrates very small round patiromer and needle-like monosodium urate crystals. (**B**) Human Caco2 and HCA7 intestinal epithelial cell lines were stimulated with patiromer (200 and 1000 µg/mL) or monosodium urate (200 and 1000 µg/mL) crystals for 24 hours. Triton served as a positive control (*n* = 5 per group). (**C**–**F**) After stimulation, culture supernatants were collected and MTT (**C,D**) as well as LDH (**E,F**) assays were performed. Cytotoxicity is presented as a percentage (%, *n* = 5 per group). Data are means ± SD. * p<0.05; ** p<0.01; *** p<0.001; ns, not significant using one-way ANOVA.

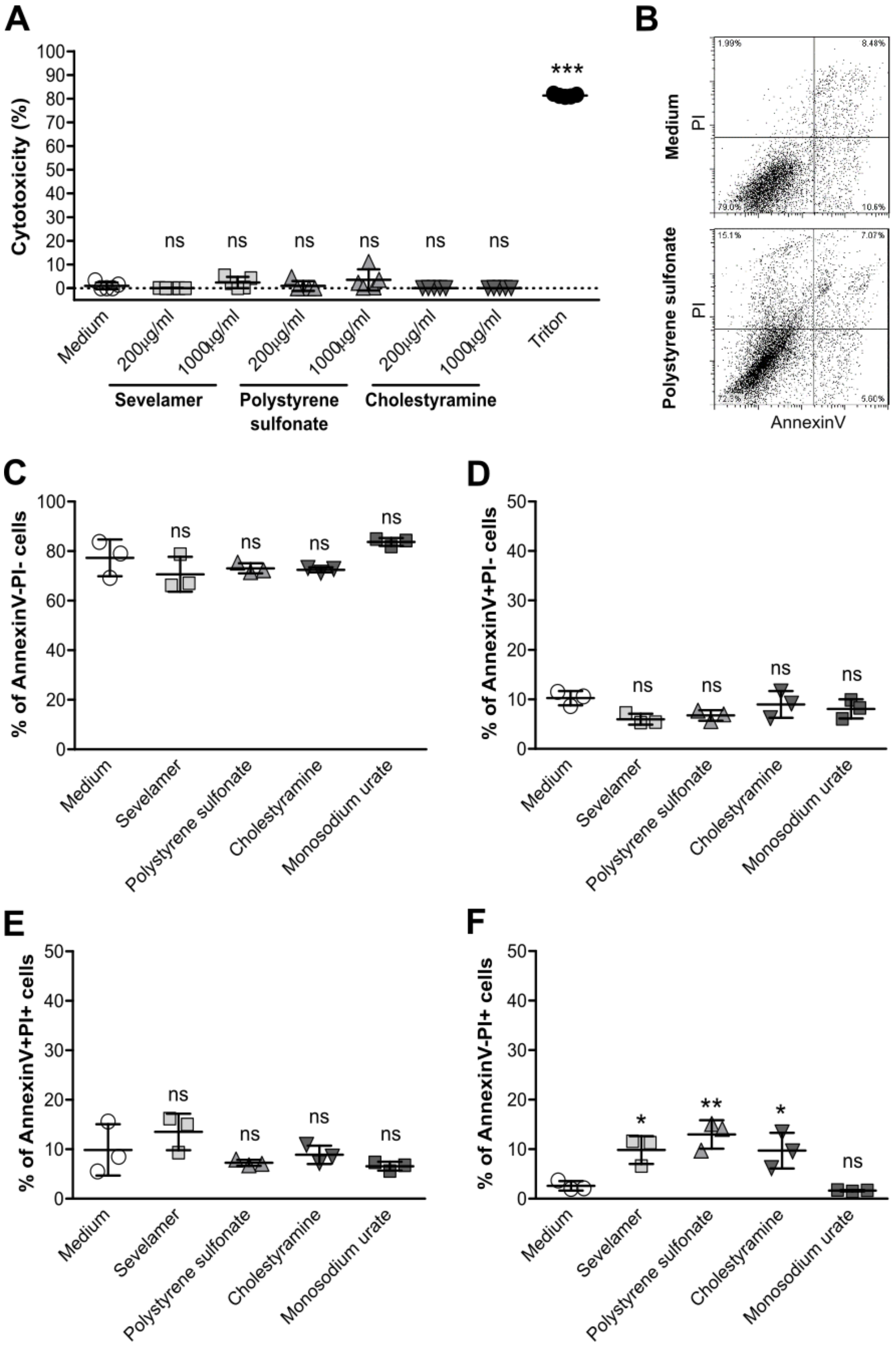

Figure S2: Drug crystals induce only necrosis in HCA7 intestinal epithelial cells. Human HCA7 colon epithelial cell lines were stimulated with different concentrations of sevelamer, polystyrene sulfonate, cholestyramine, or monosodium urate crystals (200 or 1000 µg/ml) for 24 hours. Triton was used as a positive control. (**A**) After stimulation, culture supernatants were collected and LDH assays were performed. Cytotoxicity is presented as a percentage (%, *n* = 5 per group). (**B**-**F**) AnnexinV/PI staining of HCA7 cells was performed by flow cytometry (**B**). The percentage of live (AnnexinV-PI-)(**C**), apoptotic (AnnexinV+PI-)(**D**), late apoptotic/early necrotic (AnnexinV+PI+)(**E**), and necrotic (AnnexinV-PI+)(**F**) HCA7 cells after stimulation with 1000 µg/mL of different drug crystals was quantified (*n* = 3 per group). Data are means ± SD. * p<0.05; ** p<0.01; *** p<0.001; ns, not significant versus the medium using one-way ANOVA.

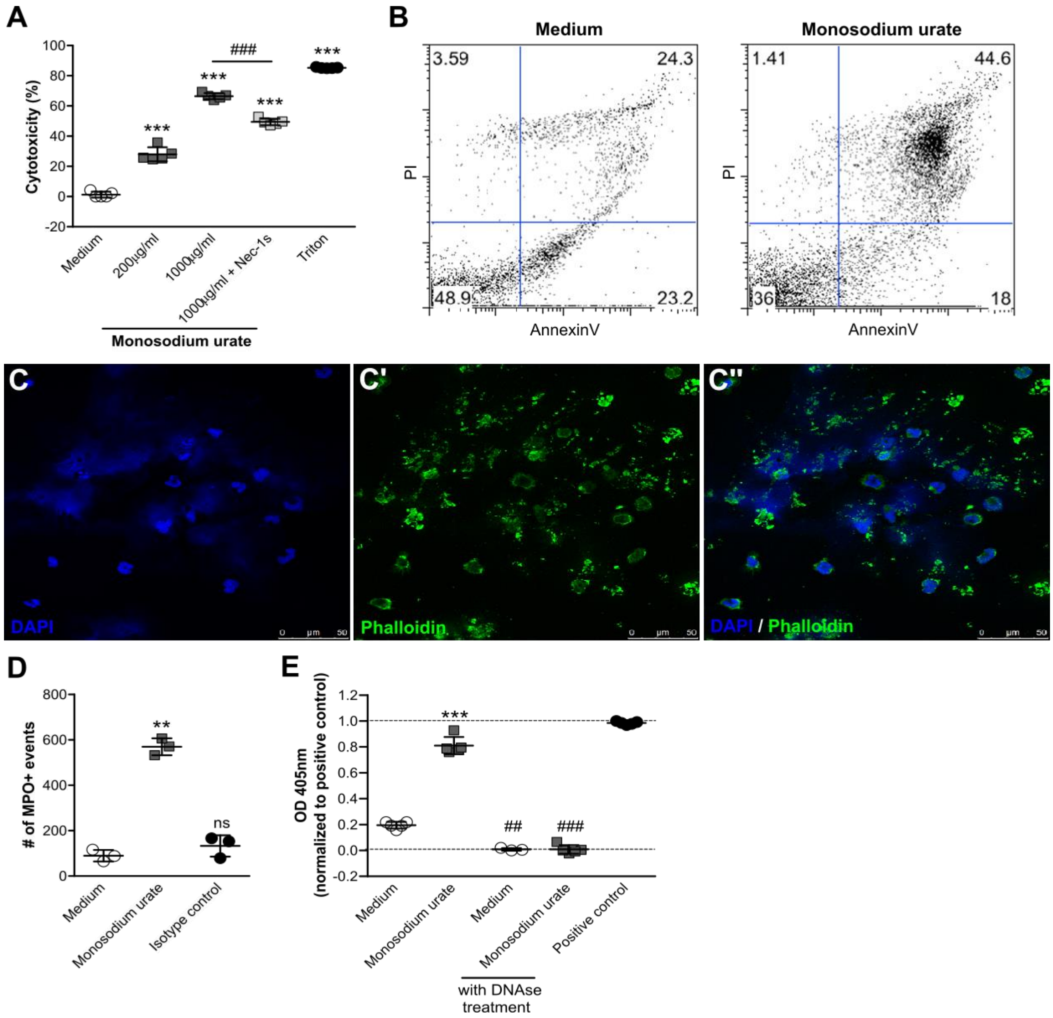

Figure S3: Monosodium urate crystals induce neutrophil extracellular trap formation**.** Human neutrophils from healthy individuals were isolated and stimulated with or without monosodium urate crystals (200 and 1000 µg/mL) for 2 hours in the absence or presence of the necroptosis inhibitor Nec-1s. Triton was used as a positive control (*n* = 5 per group). (**A**) After stimulation, culture supernatants were collected and LDH assays were performed. Cytotoxicity is presented as a percentage (%). Data are means ± SD. *** and ### p<0.001 versus medium using one-way ANOVA. (**B**) Cell death was determined by AnnexinV/PI staining of monosodium urate crystal-activated (200 µg/mL) neutrophils via flow cytometry. Representative dot plots are shown. (**C**) Neutrophils cultured with monosodium urate crystals (200 µg/mL) were stained with DAPI (blue) and phalloidin (green) for fluorescence microscopy. Images are shown as a merge of phalloidin and DAPI (200× magnification). (**D**) The number of myeloperoxidase (MPO)-positive events was determined in the supernatants of human neutrophils stimulated with or without monosodium urate (1000 µg/mL) after 2 hours by flow cytometry. (**E**) Quantification of released histone-complexed DNA fragments (OD 450 nm) in culture supernatants of crystal-stimulated neutrophils after 2 hours using a cell death detection ELISA^{PLUS} assay. DNAse treatment was used as a control. Data are means ± SD. ** p<0.01; *** p<0.001; ns, not significant (p≥0.05) versus the medium, and ## p<0.01; ### p<0.001 versus without DNAse treatment, using one-way ANOVA.

© 2020 by the authors. Licensee MDPI, Basel, Switzerland. This article is an open access article distributed under the terms and conditions of the Creative Commons Attribution (CC BY) license (http://creativecommons.org/licenses/by/4.0/).
